# Evaluation of acetone as a solvent for the Ames test

**DOI:** 10.1186/s41021-020-0143-6

**Published:** 2020-01-23

**Authors:** Tomomi Shibata, Takeshi Yamagata, Akihiro Kawade, Shoji Asakura, Naoki Toritsuka, Naoki Koyama, Atsushi Hakura

**Affiliations:** 1 Management Planning Department, Sunplanet Co., Ltd, 3-5-10 Otsuka, Bunkyo-ku, Tokyo, 112-0012 Japan; 2 Preclinical Safety Research Unit, Tsukuba R&D Supporting Division, Sunplanet Co., Ltd, 5-1-3 Tokodai, Tsukuba, Ibaraki 300-2635 Japan; 3 Global Drug Safety, Eisai Co., Ltd, 5-1-3 Tokodai, Tsukuba, Ibaraki 300-2635 Japan

**Keywords:** Acetone, Reverse mutation assay, Bacteria, Solvent, Cytotoxicity, Mutagenicity, Metabolism

## Abstract

**Background:**

Acetone is a common alternative solvent used in the Ames test when test chemicals are unstable or poorly soluble in water or dimethyl sulfoxide (DMSO). Yet, there is a very limited number of studies evaluating acetone as a solvent in the modified Ames test with preincubation (preincubation test).

**Results:**

We evaluated the acetone as a solvent for the preincubation test. Fourteen mutagens dissolved in acetone was added each to the reaction mixture at 2 different volumes (25 or 50 μL) to examine mutagenicity using bacterial test strains recommended in the Organization for Economic Cooperation and Development (OECD) test guideline 471, and compared with DMSO (100 μL). Cytotoxicity of acetone was also examined in these bacterial strains. TA1537 was most sensitive to the cytotoxicity of acetone, the degree of which was moderate and similar to DMSO in TA1537 without S9 mix. In other strains, cytotoxicity was limited to a mild degree with or without S9 mix. Cytotoxicity of acetone did not affect detection of mutagenicity of any mutagens; many of them being comparable or less mutagenic than those with DMSO.

**Conclusions:**

These findings indicate that acetone is a viable candidate as a solvent for the preincubation test in the 5 bacterial strains.

## Introduction

The Ames test is used worldwide for detecting mutagenicity of chemicals using bacterial strains [1–3]. The modified Ames test with preincubation (preincubation test) is often used, particularly in Japan, since it is generally considered to be more sensitive than the original plate incorporation method where the test article as well as other components are diluted [4, 5]. In the Guidebook for Mutagenicity Test Guideline in Industrial Safety and Health Act, water, dimethyl sulfoxide (DMSO), and acetone are the standard choices of the solvent [4].

DMSO is the most widely used organic solvent for Ames tests due to its limited cytotoxicity, and its property as a solvent to dissolve a wide variety of substances and its low reactivity with organic test chemicals except for some compounds (e.g., acyl chlorides and sulfonyl chlorides) [1, 6–8]. In the preincubation test, DMSO is added at a relatively high concentration (14% = 0.1 ml of DMSO/0.7 mL of the mixture) in the reaction mixture (consisting of phosphate buffer [PB] or S9 mix, bacterial culture, and test chemical dissolved in DMSO). DMSO is well known to produce cytotoxicity and inhibit drug metabolism during the preincubation step, resulting in reduced sensitivity in the detection of mutagens [9, 10]. We previously examined the effects of multiple concentrations of DMSO on the cytotoxicity to the bacterial strains and on the mutagenicity of mutagens; in the preincubation test, DMSO did not affect the detection of mutagens in spite of moderate cytotoxicity up to a concentration of 14% [9, 10].

When test chemicals are unstable or poorly soluble in water or DMSO, acetone is one of the primary solvent choices [4, 6]. The guidebook for Mutagenicity Test Guideline in Industrial Safety and Health Act states [4] that when test chemicals are poorly soluble in either solvent of water, DMSO, or acetone, other solvents which do not affect both bacterial viability and S9 activity can be used. In spite of its wide and preferred use, there is a very limited number of studies on evaluation of acetone as a solvent [6, 11]. The purpose of the present study is to assess acetone as a solvent in the preincubation test, including cytotoxicity and its effect on the sensitivity of detection of mutagenicity of 14 representative mutagens in the 5 bacterial strains recommended for use in the Organization for Economic Cooperation and Development (OECD) test guideline 471 [3]; the results are discussed in reference to those in 100 μL of DMSO in TA100 or multiple bacterial strains. Our data indicate that acetone is a valid alternative solvent to DMSO in the preincubation test.

## Materials and methods

### Materials

Table 1 shows the name, abbreviations, CAS No., source, and purity of the test mutagens used. Acetone (the grade of guaranteed reagents, > 99.5% purity, FUJIFILM Wako Pure Chemical Co., Osaka, Japan), dimethyl sulfoxide (DMSO, the grade of biochemistry use, 100% purity, FUJIFILM Wako Pure Chemical Co.), and water for injection (as purified water, Otsuka Pharmaceutical Factory, Inc., Tokushima, Japan) were used as solvents. Acetone was used after dehydration on molecular sieves (3 Å), which was purchased from FUJIFILM Wako Pure Chemical. Plates of minimal-glucose agar medium (TESMEDIA®AN) were obtained from Oriental Yeast Co., Ltd., Tokyo, Japan. Oxoid Nutrient Broth No.2 and Bacto agar were purchased from Oxoid Ltd., Hampshire, UK, and Becton, Dickinson & Company., Maryland, US, respectively. The S9 fraction of phenobarbital/5,6-benzoflavone-pretreated male Sprague-Dawley rat liver was purchased from Oriental Yeast, Co., Ltd. The S9 mix (0.5 mL) consisted of 0.05 mL of the S9 fraction and 0.45 mL of a cofactor solution (Cofactor-1®; Oriental Yeast Co., Ltd.), and contained 8 mM MgCl_2_, 33 mM KCl, 5 mM glucose-6-phosphate, 4 mM NADPH, 4 mM NADH and 100 mM sodium phosphate (pH 7.4).

**Table 1:** Mutagens used in this study

Chemical name	Abbreviation	CAS No.	Source	Purity
Methyl methansulfonate	MMS	66–27-3	Tokyo Chemical	> 98.0
Cyclophosphamide hydrate	CP	6055–19-2	Shionogi	JP^a^
Methyl yellow	MY	60–11-7	Tokyo Chemical	GR^b^
2-Acetylaminofluorene	2-AAF	53–96-3	Tokyo Chemical	> 98.0
Quinoline		91–22-5	Tokyo Chemical	> 98.0
7,12-Dimethylbenz [*a*]anthracene	DBA	57–97-6	Tokyo Chemical	> 98.0
2-Aminoanthracene	2AA	613–13-8	FUJIFILM Wako Pure	96.7
*N*-Nitrosopyrrolidine	NP	930–55-2	Sigma-Aldrich	> 98.0
Benzo [*a*]pyrene	BP	50–32-8	Sigma-Aldrich	> 96.0
Sodium azide	SA	26,628–22-8	FUJIFILM Wako Pure	99.9
2-Nitrofluorene	2-NF	607–57-8	Tokyo Chemical	> 99.0
9-Aminoacridine hydrochloride monohydrate	9AA	52,417–22-8	Tokyo Chemical	99.4
2-(2-Furyl)-3-(5-nitro-2-furyl)acrylamide	AF2	3688-53-7	FUJIFILM Wako Pure	> 98.0
4-Nitroquinoline 1-oxide	4NQO	56–57-5	Tokyo Chemical	> 99.0

^a^Japanese Pharmacopoeia

^b^GR: manufacturer-guaranteed reagent, and purity not specifically determined

### Bacterial strains

*Salmonella typhimurium* TA100, TA1535, TA98, and TA1537 and *Escherichia coli* WP2*uvrA* were used in this study were provided by CIMIC Bioresearch Center Co., Ltd., Yamanashi, Japan.

### Measurement of the number of bacterial surviving cells

Bacterial overnight cultures of each test strain in the early stationary phase were prepared. To each test tube containing 0.5 mL of 100 mM sodium PB (pH 7.4) or S9 mix, 0.1 mL of bacterial overnight culture and then 0 μL (no solvent), 25 μL (final solvent concentration of 4.0%), or 50 μL (7.7%) of acetone, were added. Immediately after mixing, 20 μL of an aliquot was taken from the mixture to determine the number of surviving cells just before the preincubation phase (as the control for each strain with or without S9 mix). The residual mixture was incubated with shaking (approximately 120 strokes per min) at 37 °C for 20 min. Then, 20 μL of an aliquot was taken from the mixture to determine the number of surviving cells. Each aliquot was serially diluted 10^5^-fold with PB or physiological saline, and 0.3 mL (all *Salmonella* strains) or 0.1 mL (WP2*uvrA*) of the diluted cell suspension was combined with 2.5 mL of molten nutrient broth top agar. The content was immediately poured onto a plate of minimal glucose agar medium. The plate was incubated at 37 °C for about 24 h, and the number of colonies that appeared was counted as that of survivors. Each experiment for cytotoxicity was conducted using duplicate test tubes and duplicate plates for each test tube. The experiments were conducted twice or three times (for TA1537).

### Mutagenicity test

The Ames preincubation test was conducted for mutation. The method taken before the end of the preincubation phase was similar to that employed in the cytotoxicity test. The mutagenicity test was independently conducted from the cytotoxicity test. Briefly, to each test tube containing 0.5 mL of PB or S9 mix, 0.1 mL of bacterial culture, and then 0 μL (no solvent), 25 μL (final solvent concentration of 4%), 50 μL (7.7%), or 100 μL (14.3%) of a test mutagen dissolved in either solvent (25 μL and 50 μL for acetone, 100 μL for DMSO and purified water) were added. Immediately the treatment mixture was incubated at 37 °C for 20 min with shaking. After completion, the treatment mixture was immediately mixed with 2 mL of 0.05 mM L-histidine/0.05 mM biotin molten top agar (*Salmonella* strains) or 0.05 mM L-tryptophan (WP2*uvrA*), and the content was poured onto a plate of minimal-glucose agar medium. The plate was incubated at 37 °C for approximately 48 h, and revertant colonies that appeared were counted. Sign of bacterial background lawn was also checked as an indicator of cytotoxicity. The assays were conducted twice, including a dose-finding and main assays.

## Results

### Cytotoxicity of acetone to bacteria

The reaction mixture was prepared by varying volumes of acetone added to each strain with or without S9 mix to determine cytotoxicity. The mixture was preincubated for 20 min, and an aliquot was taken for determination of cytotoxicity by determining the number of surviving cells. Table 2 shows the numbers of surviving cells of the five bacterial test strains recommended in OECD test guideline 471 (*S. typhimurium* TA100, TA1535, TA98, and TA1537 and *E. coli* WP2*uvrA*) in the presence or absence of S9 mix before and after preincubation, with either 25 or 50 μL of acetone. The survival rate is expressed as a percentage of the number of survivors compared with those without acetone in the absence of S9 mix before preincubation.

**Table 2:** Cytotoxicity of acetone on bacterial cells with or without S9 mix, before and after preincubation in the Ames preincubation test

S9 mix	Preincubation	Volume of acetone (μL)	Number of survivors (× 10^8^ cells/mL)				
			TA100	TA1535	TA98	TA1537	WP2*uvrA*
without	Before	0	4.13 ± 0.24	5.48 ± 0.22	4.90 ± 0.28	4.84 ± 0.24	3.02 ± 0.32
			(100)	(100)	(100)	(100)	(100)
		25	4.08 ± 0.38	5.86 ± 0.15	4.69 ± 0.23	4.87 ± 0.20	ND
			(99)	(107)	(96)	(101)	ND
		50	3.99 ± 0.23	5.52 ± 0.21	4.70 ± 0.19	4.80 ± 0.16	2.71 ± 0.20
			(97)	(101)	(96)	(99)	(90)
	After	0	4.32 ± 0.16	6.02 ± 0.27	5.31 ± 0.21	4.56 ± 0.22	2.89 ± 0.10
			(105)	(110)	(108)	(94)	(96)
		25	3.71 ± 0.24	5.50 ± 0.15	4.58 ± 0.13	4.03 ± 0.37	ND
			(90)	(100)	(93)	(83)	ND
		50	2.70 ± 0.28	4.25 ± 0.26	4.29 ± 0.20	3.83 ± 0.18	2.86 ± 0.14
			(65)	(78)	(88)	(79)	(95)
with	Before	0	3.99 ± 0.22	5.29 ± 0.20	4.28 ± 0.26	3.51 ± 0.21	2.42 ± 0.53
			(96)	(97)	(87)	(73)	(80)
		25	3.81 ± 0.23	4.87 ± 0.18	4.66 ± 0.16	3.21 ± 0.16	2.69 ± 0.23
			(92)	(89)	(95)	(66)	(89)
		50	3.49 ± 0.16	4.70 ± 0.23	4.36 ± 0.33	3.04 ± 0.29	2.61 ± 0.25
			(84)	(86)	(89)	(63)	(86)
	After	0	3.81 ± 0.25	5.01 ± 0.38	3.69 ± 0.28	2.23 ± 0.19	3.01 ± 0.66
			(92)	(91)	(75)	(46)	(100)
		25	2.93 ± 0.13	3.92 ± 0.29	3.58 ± 0.14	1.56 ± 0.12	2.34 ± 0.60
			(71)	(72)	(73)	(32)	(78)
		50	1.80 ± 0.07	2.78 ± 0.11	1.99 ± 0.11	1.16 ± 0.17	2.46 ± 0.18
			(44)	(51)	(41)	(24)	(81)

ND; Not determined. The number of survivors was determined in a treatment mixture containing different amounts of acetone and 0.1 mL of bacterial culture with or without 0.5 mL of S9 mix, before or after preincubation at 37 °C for 20 min. The number of survivors indicated is the mean ± standard deviation of those obtained from duplicate test tubes and duplicate plates for each test tube. Figures in parentheses are ratio of the number of survivors expressd as a percentage of the number of survivors for those without acetone in the absence of S9 mix before preincubation

Addition of 25 μL of acetone to a test tube containing 0.5 mL of PB and 0.1 mL of bacterial culture had no or minimal cytotoxicity (the survival rates were 83 to 100%) in any strain without S9 mix. In the presence of S9, acetone was mildly cytotoxic (the survival rates were 71 to 78%) in TA100, TA1535, TA98, and WP2*uvrA*. When 50 μL of acetone was added in the absence of S9 mix, no or minimal cytotoxicity (the survival rates were 78 to 95%) was observed in TA1535, TA98, and WP2*uvrA*, with mild cytotoxicity (the survival rate was 65%) in TA100. In the presence of S9, there was minimal to moderate cytotoxicity (the survival rates were 41 to 81%) in TA100, TA1535, TA98, and WP2*uvrA*.

TA1537 was most sensitive to the cytotoxicity of acetone among all strains tested; the cytotoxic effect was minimal (the survival rates; 83% for 25 μL and 79% for 50 μL) in the absence of S9 mix, whereas increased to a moderate degree (the survival rates; 32% for 25 μL and 24% for 50 μL) in its presence. A decrease in the number of survivors of TA1537 in the presence of S9 mix was observed before preincubation (73% survival), but not in the absence of S9 mix, suggesting that the cytotoxicity of acetone was increased upon exposure to S9 mix. The experiment for cytotoxicity was carefully repeated; 10^5^-diluted suspension for plating was immediately prepared using an aliquot from the reaction mixture because there was a possibility that acetone metabolites generated by remaining (contaminating) S9 mix were cytotoxic. Hence, the data shown in Table 2 is considered to be valid. A trend for a decrease in bacterial viability, particularly for TA1537, in the presence of S9 mix before preincubation was consistent with that in our previous study [9]. Although the cause for that trend is not clear, it is speculated to be due to activated oxygen species generated by microsome in the S9 mix.

### Effect of acetone on the mutagenicity of mutagens (50 μL of acetone solution *v.s.* 100 μL of DMSO solution)

We examined the effect of acetone (addition of 25 μL or 50 μL of solution, which is often used) on the mutagenicity of 14 representative mutagens. The mutagenicity of these mutagens dissolved in DMSO (100 μL, which is often used) was also examined for comparison. MMS is a direct mutagen that does not require metabolic activation [12, 13]. CP, MY, 2-AAF, Quinoline, DBA, 2AA, NP, and BP are promutagens that require CYP enzymes contained in S9 mix for metabolic activation [10, 13, 14]. SA, 2-NF, 9AA, AF2, and 4NQO are promutagens that are metabolically activated by bacterial nitroreductase enzymes [10, 13, 14].

**Fig. 1:**
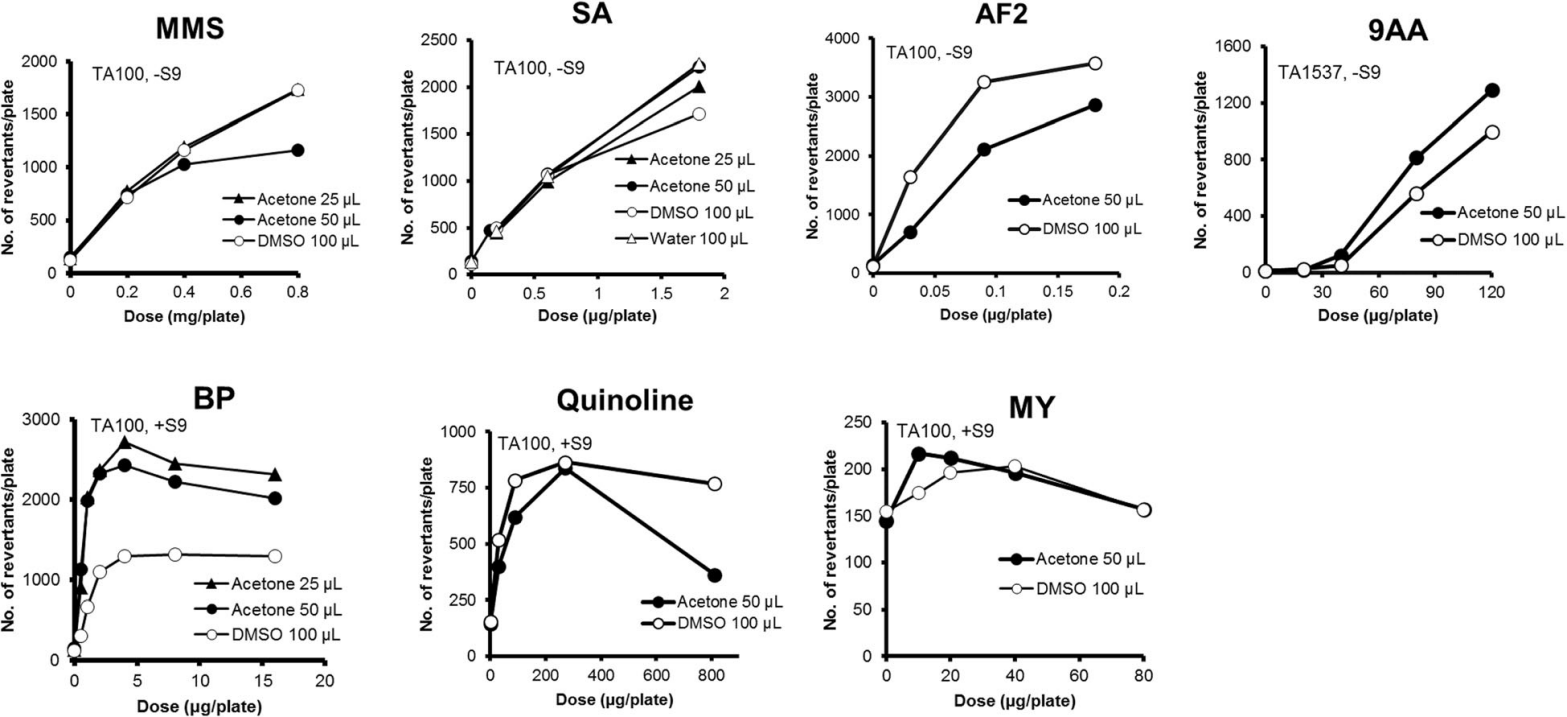
The mutagenicity of mutagens performed with a single strain in the presence of 25 μL of acetone (▲), 50 μL of acetone (●), 100 μL of DMSO (○), and 100 μL of aqueous solutions (△)

**Fig. 2:**
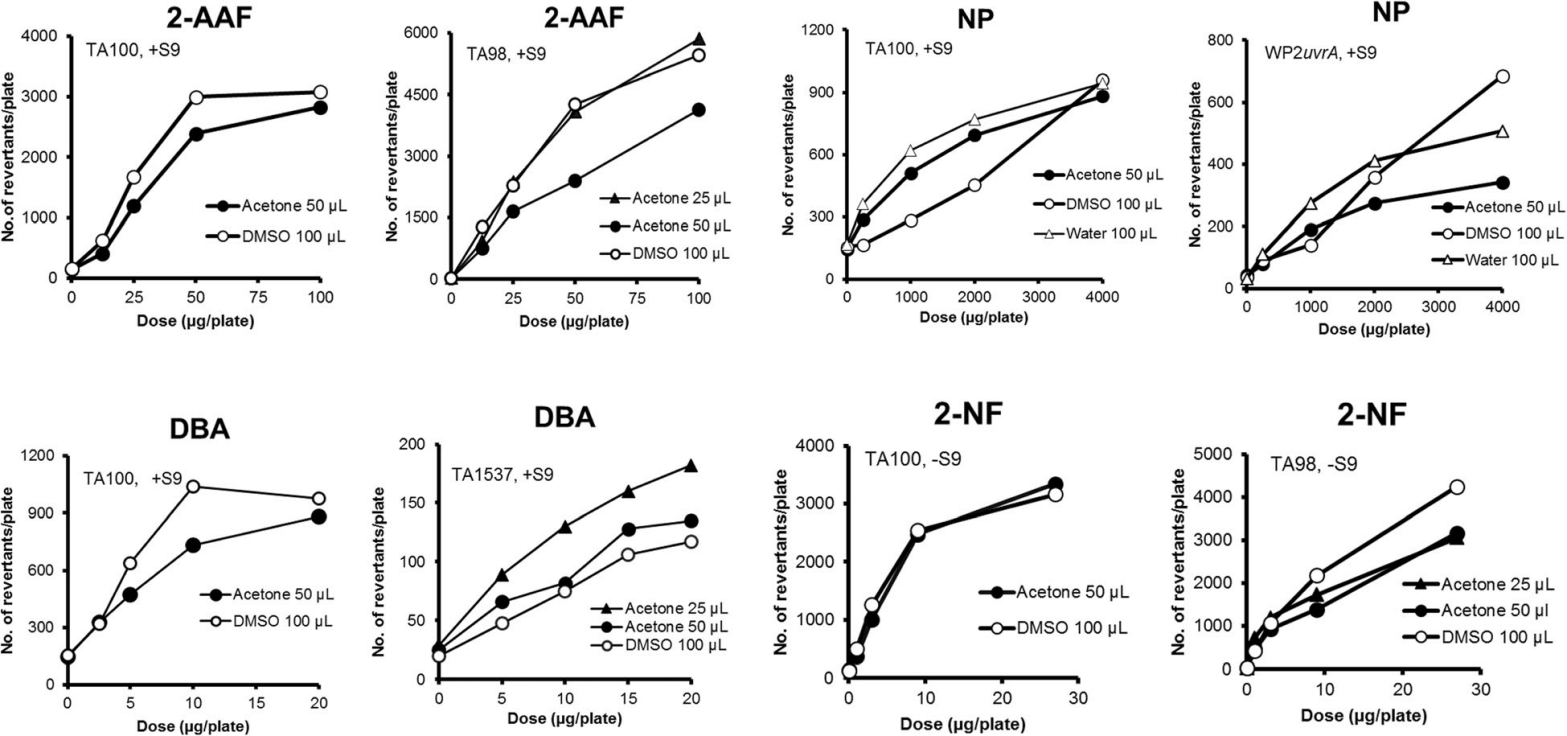
The mutagenicity of mutagens performed with 2 strains in the presence of 25 μL of acetone (▲), 50 μL of acetone (●), 100 μL of DMSO (○), and 100 μL of aqueous solutions (△)

**Fig. 3:**
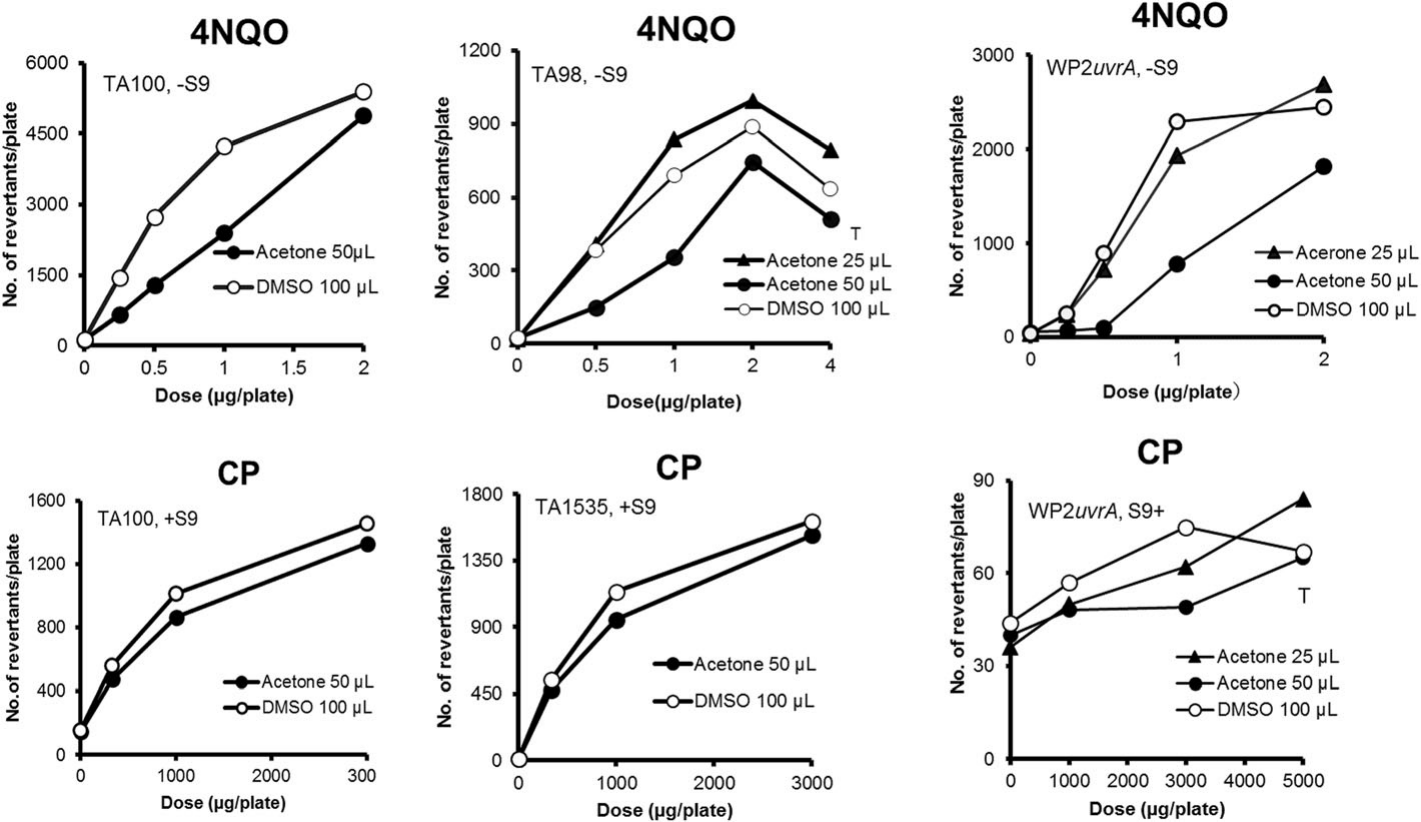
The mutagenicity of mutagens performed with 3 strains in the presence of 25 μL of acetone (▲), 50 μL of acetone (●), and 100 μL of DMSO (○) solutions. The symbol “T” indicates toxic (reduced bacterial background lawn)

**Fig. 4:**
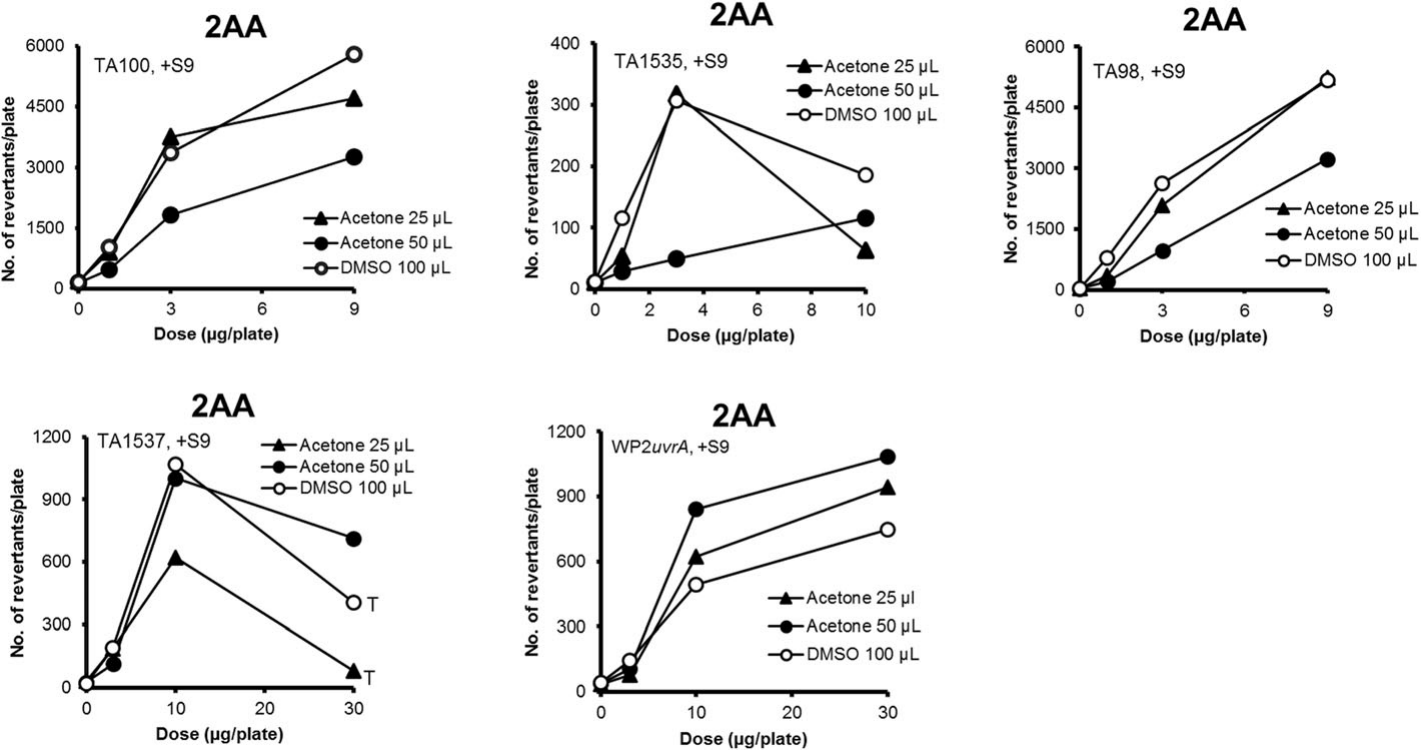
The mutagenicity of mutagens performed with multiple strains in the presence of 25 μL of acetone (▲), 50 μL of acetone (●), and 100 μL of DMSO (○) solutions. The symbol “T” indicates toxic (reduced bacterial background lawn)

There were no differences in the mutagenicity between 50 μL of acetone solution and 100 μL of DMSO solution for MMS, SA, CP, MY, 2-AAF, 2-NF, quinoline, and DBA in TA100 (Figs. 1, 2 and 3). On the other hand, moderate differences in the mutagenicity were found with AF2, 4NQO, BP, 2AA, and NP in TA100 (Figs. 1, 2, 3 and 4), and with 2-AAF in TA98 (Fig. 2); the mutagenicity of AF2, 4NQO, 2-AAF and 2AA, dissolved in acetone (50 μL) were weaker than those dissolved in DMSO (100 μL) (Figs. 1, 2, 3 and 4), while the mutagenicity of BP and NP dissolved in acetone were more potent than that dissolved in DMSO (Figs. 1 and 2). Why mutagenicity of BP or NP was more potent when dissolved in acetone than in DMSO is not clear. BP and NP are metabolically activated or detoxified in different, parallel or sequential pathways by CYP enzymes [14]; inhibition of detoxification by DMSO is speculated to be more powerful than acetone, or alternatively inhibition of activation by acetone was weaker than DMSO. The inhibition of enzyme activities by small molecule organic solvents including acetone as well as DMSO is well-documented [15–18].

### Comparison of the mutagenicity of mutagens (25 μL *v.s.* 50 μL of acetone solutions)

When we compared the mutagenicity of mutagens with addition of either 25 or 50 μL of acetone solution, there were no differences in the mutagenicity of MMS, SA and BP in TA100 (Fig. 1), and CP and 2AA in WP2*uvrA* (Figs. 3 and 4), and 2-NF in TA98 (Fig. 2). On the basis of the results for 14 mutagens tested, the mutagenicity induced by addition of 25 μL of acetone solution was considered to be equivalent to 100 μL of DMSO solution, whereas the mutagenicity induced by addition of 50 μL of acetone solution was weaker than addition of 100 μL of DMSO solution, as previously stated. However, the difference was small, and the addition of either 25 or 50 μL of acetone solution was considered adequate with the preincubation test with or without S9 mix in all of the 5 strains tested. One possible reason for the different degrees of mutagenicity observed with promutagens in 25 μL and 50 μL of acetone solutions, and 100 μL of DMSO solution is the differences in the degree of inhibition of multi-step sequential or parallel activation or detoxification by P450 subfamilies or bacterial nitroreductase enzymes [13, 14].

### Comparison of mutagenicity of mutagens by addition of 25 or 50 μL of acetone solutions, or 100 μL of DMSO or aqueous solutions among multiple strains: strain differences

Mutagens dissolved in acetone (25 or 50 μL), DMSO (100 μL), or purified water (100 μL) were examined for mutagenicity in multiple strains. Mutagens tested include 2AA, 2-AAF, NP, 2-NF, CP, 4NQO, and DBA.

The mutagenicity of 2AA was detected in three different patterns of mutagenicity (Fig. 4). 2AA showed similar mutagenic activity in TA100, TA1535, and TA98 with 25 μL of acetone solution and 100 μL of DMSO solution, with less mutagenic activity in the presence of 50 μL of acetone solution. In TA1537, mutagenicity in the presence of 50 μL of acetone solution and 100 μL of DMSO solution were similar, with less mutagenicity in the presence of 25 μL of acetone solution. In WP2*uvrA*, mutagenicity by addition of 25 μL and 50 μL of acetone solutions were roughly equal to that by 100 μL of DMSO solution. The effects of acetone on the mutagenicity of 2AA at the volumes of 25 μL and 50 μL were different among the strains. Mutagenicity of 2AA is more efficiently detected at a lower amount of S9 fraction than 50 μL (amount commonly used for S9 fraction in a test tube) [10, 19], suggesting that when the concentration of S9 fraction is high, detoxification pathways is dominant. The magnitude of mutagenicity at varying volumes of acetone in different bacterial strains, would reflect mutations from different types and amounts of DNA adducts. It will be determined by the degree of inhibition of activation and detoxification pathways. The inhibitory effects in TA1537 may be different from other strains.

2-AAF showed a different pattern of the mutagenicity in TA100 and TA98; the mutagenicity in TA100 with 50 μL of acetone solution and 100 μL of DMSO solution was similar, while the mutagenicity in TA98 with 100 μL of DMSO solution was more potent than that with 50 μL of acetone solution (Fig. 2). NP showed a different pattern of mutagenicity between TA100 and WP2*uvrA*; the mutagenicity with 50 μL of acetone solution was more potent than that with 100 μL of DMSO solution in TA100, while the mutagenicity with 50 μL of acetone solution was less than 100 μL of DMSO solution (Fig. 2).

On the contrary, for 2-NF, CP, 4NQO, and DBA, there were no differences in the pattern of the mutagenicity; in the presence of 50 μL of acetone solution and 100 μL of DMSO solution, mutagenicity was similar among bacterial strains (2-NF; TA100 and TA98, CP; TA100, TA1535, and WP2*uvrA*, DBA; TA100 and TA1537) (Figs. 2 and 3). There were no differences in the pattern of mutagenicity; the mutagenicity of 4NQO in the presence of 100 μL of DMSO solution was more potent than 50 μL of acetone solution in TA100, TA98, and WP2*uvrA*.

These findings indicate that different strains showed different responses to each mutagen, depending on the solvents and the volume added. These bacterial strains harbor different mutations [1, 2], and each strain probably detected mutations resulting from different types and amounts of DNA adducts generated by different inhibition of metabolism for each mutagen [15–18].

### Cytotoxic effect of acetone on the mutagenicity by mutagens in TA1537

As previously stated, addition of 50 μL of acetone decreased the TA1537 survival rate to 24% in the presence of S9 mix (Table 2). We tested the mutagenicity of three mutagens (9AA, DBA, and 2AA) dissolved in acetone using TA1537 to examine if the cytotoxic effect of acetone influences their mutagenicity. As shown in Figs. 1, 2 and 4, the mutageniciy of 9AA, DBA, and 2AA in the presence of 50 μL of acetone solution was equivalent to 100 μL of DMSO solution. No reduction of bacterial background lawn was noted in any plates after addition of 25 or 50 μL of acetone solution, or 100 μL of DMSO solution except for only at higher doses, where reduced numbers of revertant colonies were found (4NQO with TA98, CP with WP2*uvrA*, and 2AA with TA1537). This finding indicates that up to 50 μL of acetone solution did not affect the detection of these mutagens in the preincubation test in spite of moderate cytotoxicity.

## Discussion

We evaluated the use of acetone in the preincubation test. The effect was limited to moderate cytotoxicity in the bacterial strains recommended for use in OECD test guideline 471 [3]. The presence of S9 mix increased the cytotoxicity. The magnitude of cytotoxicity of acetone was dependent on the bacterial strains, with TA1537 being the most sensitive strain (the survival rate reduced to 24% in the presence of S9 mix). The degree of mild cytotoxicity of 50 μL acetone was comparable to that of 100 μL DMSO. No reduction of bacterial background lawn was noted after addition of 25 or 50 μL of acetone solutions. In spite of such cytotoxic effect of acetone, mutagenicitiy of 14 representative mutagens was detected with either 25 or 50 μL of acetone solution, as with 100 μL of DMSO solution. For many of mutagens examined, the mutagenicity in the presence of 25 μL of acetone solution was similar to those with 100 μL of DMSO solution, and more sensitive than 50 μL of acetone solution. Acetone, a small molecule organic solvent is well known to inhibit drug-metabolizing enzymes [15–18] as DMSO. Our results with acetone (solvent control) and mutagens (positive control) used in this study were consistent with literature values for each strain [20]. The paper [21] published by the 2017 International Workshop on Genetic Toxicology Testing (IWGT) advices that the solvent should be used in a way which does not lower bacterial viability or lower S9 activity. The present study provides evidence that the use of acetone was a viable alternative choice of the solvent in the preincubation test.

One possible reason why acetone is not as frequently used as a solvent may be to do with the fact that formulation prepared is prone to drip from the pipette tips at the time of dispensing; this is due to a high vapor pressure/low boiling point (56.5 °C) of acetone [22]. The dripping of formulation is easily avoided by filling the pipette with the vapor just before dispensing the formulation by drawing acetone in and out of the pipette a few times.

## Conclusion

Acetone was validated as a solvent for the Ames test in mutagenicity test with multiple mutagens and cytotoxicity test, using the standard bacterial strains. Our data provide strong evidence to support the use of acetone up to a volume of 50 μL as a valid alternative solution in the Ames preincubation test when test chemicals are unstable or poorly soluble in water or DMSO.

## Data Availability

The datasets generated and analyzed during the current study are available from the corresponding author on reasonable request.
